# Anti-inflammatory treatment of depression: study protocol for a randomised controlled trial of vortioxetine augmented with celecoxib or placebo

**DOI:** 10.1186/s13063-018-2829-7

**Published:** 2018-08-20

**Authors:** Célia Fourrier, Emma Sampson, Natalie T. Mills, Bernhard T. Baune

**Affiliations:** Department of Psychiatry, Melbourne Medical School, The University of Melbourne, Parkville, 3010 VIC Australia

**Keywords:** Major depressive disorder, Inflammation, C-reactive protein, Antidepressant, Anti-inflammatory medication, Vortioxetine, Celecoxib, Randomised controlled trial

## Abstract

**Background:**

In patients with major depressive disorder (MDD), antidepressant response and remission rates are low, highlighting the need for new treatment approaches. Recently, the abundant literature linking inflammatory processes and depressive symptoms have led to the hypothesis that selecting treatment for MDD based on the patient’s inflammatory status could be a promising strategy to improve outcomes in patients suffering from MDD. The aim of the randomised control trial we propose is to investigate the antidepressant efficacy of the combined treatment of MDD with antidepressant medication plus anti-inflammatory medication in individuals with raised inflammation levels. For the first time, this study will prospectively test the efficacy of an antidepressant plus anti-inflammatory augmentation based on baseline inflammatory maker levels in MDD using a randomised controlled trial design.

**Methods:**

This study proposes to measure blood C-reactive protein (CRP) levels before the initiation of treatment in 200 participants with MDD. Study participants are then assigned into one of two study strata: either into the ‘Depression with inflammation’ stratum (CRP levels > 3 mg/L); or into the ‘Depression without inflammation’ stratum (CRP levels ≤ 3 mg/L). Within each of the two study strata, participants randomly receive either antidepressant medication alone (vortioxetine) plus anti-inflammatory medication (celecoxib) or vortioxetine plus placebo for six weeks. At the end of the treatment period, participants have the opportunity to continue vortioxetine alone for a six-month post-trial period. Clinical outcomes are measured at baseline, fortnightly during the treatment period and at the three-month and six-month post-trial visits. The primary outcome is change in MADRS score, with a primary endpoint of a score reduction by 50% from baseline to six weeks (end of augmentation treatment with celecoxib). Secondary clinical outcomes are changes in the cognitive dimensions of depression (cognitive function, emotion processing and social cognition). Biological outcome measures (levels of CRP and other inflammatory markers) are measured at baseline, after six weeks of treatment and at the six-month post-trial visit.

**Discussion:**

The current study will generate novel evidence for biomarker-based personalised antidepressant treatment selection based on patient inflammatory status before treatment.

**Trial registration:**

Australian New Zealand Clinical Trials Registry (ANZCTR), ACTRN12617000527369p. Registered on 11 April 2017.

**Electronic supplementary material:**

The online version of this article (10.1186/s13063-018-2829-7) contains supplementary material, which is available to authorized users.

## Background

It is estimated that antidepressant medications may have therapeutic efficacy in as few as one-third of patients with major depressive disorder (MDD) [1, 2]. Furthermore, antidepressant treatment is currently selected on the basis of clinical symptoms only, including reported symptoms and observable mental state changes, making it difficult for the clinician to select the right treatment.

Several studies have highlighted a role of inflammatory processes in the development of depressive symptoms in a subgroup of patients with MDD. The central nervous system (CNS) has long been considered an immune-privileged organ, but abundant evidence documents bi-directional communication with the peripheral immune system [3]. Inflammatory cytokines released peripherally by immune cells can reach the brain through humoral, nervous and chemical pathways and hence act within the CNS [4, 5]. In the brain, inflammatory cytokines lead to the activation of glial cells (i.e. microglia and astrocytes) and coordinate a set of behavioural changes referred to as sickness behaviour in sick individuals [6–8]. Cytokines are strong modulators of several biological systems involved in behavioural regulation, such as mood regulation [6, 9].

In the context of chronic inflammation, sustained brain immune cell activation and cytokine production can lead to the development of neuropsychiatric symptoms. Indeed, pro-inflammatory mediators can induce depressive symptoms, with up to 80% of interferon-α (IFN-α) treated patients suffering from mild to moderate depressive symptoms [10–14]. Interestingly, these symptoms can be prevented or treated with antidepressants [11, 15, 16]. Numerous studies have consistently associated inflammation and somatic diseases comprising inflammatory processes, such as infections and autoimmune diseases, with an increased risk of depression [17–22]. In addition, studies have consistently shown increased levels of pro-inflammatory markers among individuals with depression, particularly during acute phases [23–25]. Meta-analyses have gathered large evidence, particularly with C-reactive protein (CRP) [25, 26], interleukin-6 (IL-6) [23–27], tumour necrosis factor-α (TNF- α) [23, 24, 27] and IL-1 receptor antagonist (IL-1ra) [24–26] being elevated among patients with depression. Interestingly, levels of IL-6 decreased after treatment of the acute depressive episode [24]. Also of note is a recent brain imaging study, which found increased microglial activation, indicating neuroinflammation, among 20 individuals suffering an active depressive episode compared to 20 healthy matched controls [28].

Since depression is a very heterogeneous disorder, it is noteworthy that recent studies have suggested that inflammatory components may be used to characterise a specific subgroup of patients with depression. Baseline increased levels of pro-inflammatory markers such as CRP have been linked to greater depressive symptom severity in general [25, 29–33] and also to greater severity of symptoms, such as mood symptoms, interest, activity, suicidality and cognitive symptoms of MDD [30]. In addition, in a retrospective analysis of a clinical trial using the TNF-α blocker infliximab to treat depressive symptoms, it was suggested that moderately increased levels of CRP at baseline might be indicative of response to infliximab; however, the study was preliminary with a small sample size of 60 patients and the conclusion was based on 13 patients showing response and 11 patients showing no response to infliximab [34]. However, the proof of concept that CRP levels at baseline might be indicative of treatment response to anti-inflammatory treatments in depression has not been studied prospectively yet. This approach may help to identify those individuals who may benefit from a targeted treatment approach, potentially leading to more tailored and more effective treatment in groups of MDD patients. Moreover, a recent systematic review and meta-analysis of clinical trials recommended that the field now needs randomised controlled trials (RCTs), using depression as the primary outcome, in individuals with high inflammation levels [35]. This review also noted that targeting inflammatory markers would be a move towards novel, effective and personalised treatment of depression. Recent work from our group has addressed the issue of selecting treatment for MDD based on inflammation status. Two of these publications found emerging evidence that anti-inflammatory evidence may need to be tailored based on the patient’s inflammatory profile [36, 37]. In other recent work from our group, a systematic review of the efficacy of non-steroidal anti-inflammatory drugs (NSAIDs) in depression concluded that translation of using effective anti-inflammatory treatment has failed so far and that further work, such as the clinical trial we propose, is urgently needed to translate this potentially significant treatment to clinical practice and improve patients’ mental health outcomes in depression [38].

We therefore propose that testing CRP levels in a blood test before the commencement of treatment in patients with MDD will give clinicians an informative biomarker at hand to guide the selection of effective antidepressant treatment. CRP was chosen as the informative inflammatory marker not only because it can predict improvement in depressive symptoms following anti-inflammatory treatment [34] but also because CRP detection in the blood can be routinely done in pathology services and hence shows potentially high clinical utility. Vortioxetine was chosen as the antidepressant medication; since it has a recently obtained Therapeutic Goods Administration (TGA) indication in Australia for the treatment of MDD, it is more likely to be useful in severe depression [39] and is likely to be useful in enhancing the remittance of cognitive symptoms of depression [40]. It will be novel to investigate its potential immune-modulatory effects in secondary analyses. Celecoxib was chosen as the trial anti-inflammatory medication because of its ability to increase the efficacy of antidepressant medication when used as an augmentation strategy [41, 42]. This is a first adequately powered, RCT that prospectively tests the efficacy of an antidepressant plus adjunctive anti-inflammatory treatment based on baseline CRP levels in MDD. This approach will inform real-world treatment of depression based on a widely available blood test.

## Methods and design

### Objectives

The primary objective of this study is to investigate the antidepressant efficacy, measured by Montgomery–Åsberg Depression Rating Scale (MADRS) score, of the six-week combined treatment of MDD with antidepressant medication (vortioxetine) plus anti-inflammatory medication (celecoxib). We hypothesise that a higher efficacy and larger reduction in symptoms of depression will be observed after six weeks when treatment is combined (antidepressant and anti-inflammatory) compared to the treatment with antidepressant alone, in individuals with baseline inflammation (i.e. with baseline CRP levels > 3 mg/L). In addition, we hypothesise that no difference between treatments will be observed in individuals with no baseline inflammation (i.e. with baseline CRP levels < 3 mg/L) (see Table 1).

**Table 1:** Objectives and hypotheses of the study. Table stating study objectives and hypothesis in a generic way and in a drug-specific way

	Generic way	Drug-specific way
Primary objective	To investigate the antidepressant efficacy of the 6-week combined treatment of MDD with antidepressant plus anti-inflammatory	To investigate the antidepressant efficacy of the 6-week combined treatment of MDD with vortioxetine plus celecoxib 400 mg
Primary hypothesis	A higher efficacy and larger reduction in symptoms of depression will be observed after 6 weeks when treatment is combined (antidepressant and anti-inflammatory) in individuals with baseline inflammation	A higher efficacy and larger reduction in symptoms of depression will be observed after 6 weeks when treatment is combined (vortioxetine and celecoxib 400 mg) in individuals with baseline inflammation
Secondary objective 1	To investigate the anti-inflammatory effects of a combined treatment of MDD with antidepressant and anti-inflammatory in individuals with baseline inflammation (CRP levels > 3 mg/L) over a 6-week period	To investigate the anti-inflammatory effects of a combined treatment of MDD with vortioxetine and celecoxib 400 mg in individuals with baseline inflammation (CRP levels > 3 mg/L) over a 6-week period
Secondary hypothesis	A larger reduction of circulating levels of the inflammatory marker CRP will be observed in the group of depressed patients who are treated with antidepressant plus anti-inflammatory medication	A larger reduction of circulating levels of the inflammatory marker CRP will be observed in the group of depressed patients who are treated with vortioxetine plus celecoxib 400 mg
Secondary objective 2	To test whether there is evidence of a different effect of the combined treatment in the two inflammation strata	To test whether there is evidence of a different effect of the combined treatment with vortioxetine and celecoxib 400 mg in the two inflammation strata
Secondary objective 3	To investigate the safety of the anti-inflammatory treatment when combined with antidepressant	To investigate the safety celecoxib 400 mg treatment when combined with vortioxetine
Secondary objective 4	To investigate the effects of the antidepressant on cognition, psychosocial and workplace performance measures in MDD	To investigate the effects of vortioxetine on cognition, psychosocial and workplace performance measures in MDD

The secondary objectives of the study are:

1. To investigate the anti-inflammatory effects of a combined treatment of MDD with vortioxetine and celecoxib in individuals with baseline inflammation (CRP levels > 3 mg/L) over a six-week period. We aim to demonstrate a larger reduction of circulating levels of the inflammatory marker CRP in the group of depressed patients who are treated with antidepressant plus anti-inflammatory medication (see Table 1).
2. To test whether there is evidence of a different effect of the combined treatment in the two inflammation strata, although the study is not powered to detect this effect (see Table 1).
3. To investigate the safety of the anti-inflammatory treatment of celecoxib 400 mg when combined with vortioxetine (see Table 1).
4. To investigate the effects of vortioxetine on cognition (using the THINC-It tool) and on psychosocial and workplace performance measures in MDD (see Table 1).

### Recruitment and study design

A total of 200 patients with MDD will be included in the study. Recruitment and treatment take place in the wider Central Adelaide Local Health Network (CALHN), with all participant appointments being held in the Clinical Research Facility in the Health and Medical Sciences Building, Discipline of Psychiatry, University of Adelaide. In addition to this, and to ensure that required numbers of study participants are recruited, people from the general population who meet the inclusion criteria are able to engage with the proposed study, as it is made available through advertisement. Participants are free to withdraw from the study at any time.

**Fig. 1:**
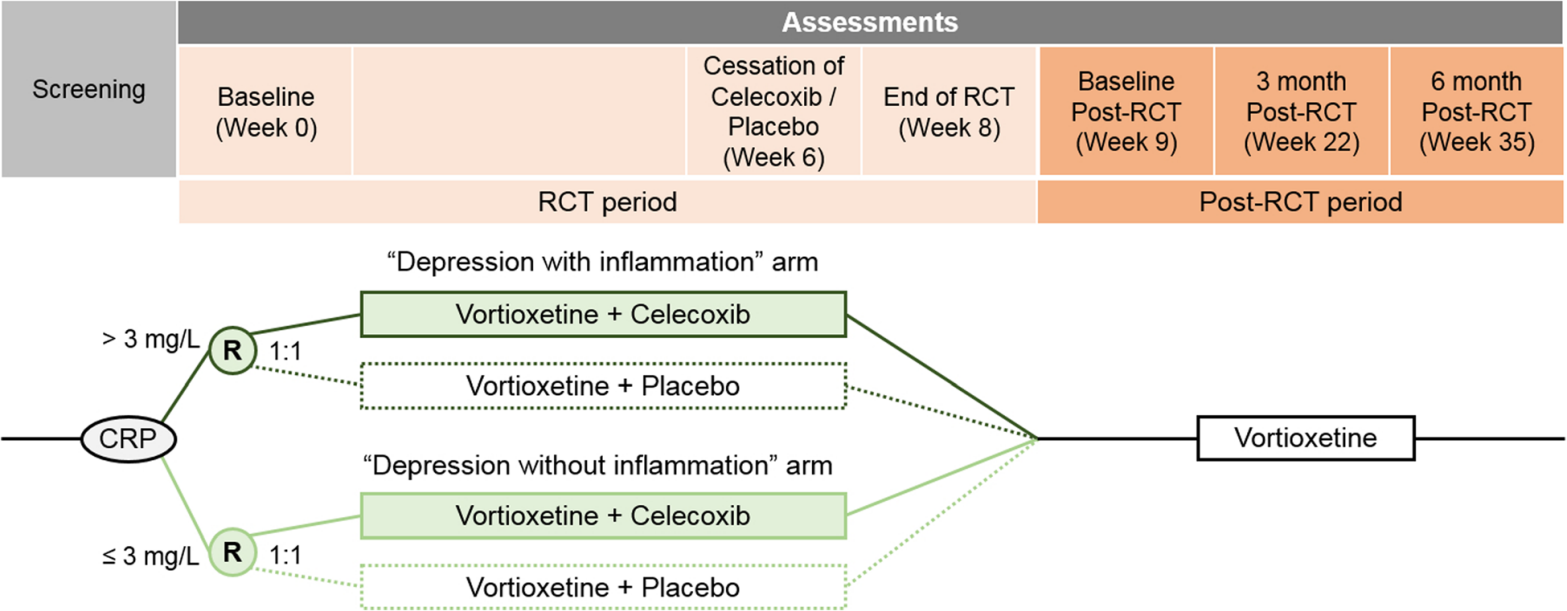
Clinical timeline of the trial. Participants will complete assessment sessions fortnightly over the 8-week RCT period. “R” circles represent 1:1 random assignment of participants within each study stratum

The study is designed as a parallel group RCT with a superiority framework, investigating 200 participants with MDD over six weeks in the primary phase followed by a six-month post-RCT follow-up period (Fig. 1). The study proposes to measure the inflammatory marker CRP before the commencement of treatment. Following CRP measurement, study participants are then assigned into one of two study strata based on CRP levels. The first of these study strata is made up of 100 individuals with CRP levels > 3 mg/L (the ‘Depression with inflammation’ stratum) and the second study stratum is an active control, made up of 100 individuals with CRP levels ≤ 3 mg/L (the ‘Depression without inflammation’ stratum). In each stratum, participants are randomly allocated in a 1:1 ratio to augmentation of vortioxetine antidepressant treatment with either celecoxib or placebo. Following six weeks of concomitant administration of celecoxib or placebo with vortioxetine, the celecoxib or placebo is then stopped and the participants are treated with vortioxetine only for an additional two weeks. This allows the opportunity to assess the efficacy of taking vortioxetine alone. While the primary time point is the six weeks in which we compare vortioxetine with augmentation against vortioxetine without augmentation, following the RCT phase, all participants have the opportunity to continue on vortioxetine without celecoxib or placebo for another six months during a post-RCT observational study period.

The principal investigator (PI) and trial psychiatrist are aware which study participants are receiving celecoxib and which participants are receiving placebo, so that the dose of vortioxetine can be determined. An approximately twofold increase in vortioxetine plasma concentration has been observed following co-administration of bupropion, a strong CYP2D6 inhibitor [43]. When bupropion was added to vortioxetine 10 mg daily at steady state, a higher incidence of adverse events was observed than when vortioxetine was added to bupropion at steady state. This may be explained by pharmacodynamics or combined pharmacodynamic/pharmacokinetic effects following CYP2D6 inhibition with bupropion, which seem to be more pronounced when the inhibition is initiated when vortioxetine is in steady state than before vortioxetine is administered [43]. In the study where bupropion was added to vortioxetine, increased incidences of nausea, headache and vomiting were noted [43]. As celecoxib is also a strong inhibitor of CYP2D6, individuals receiving celecoxib start at a lower dose of vortioxetine, 5 mg daily, and are restricted to a maximum dose of 10 mg daily for the duration of co-administration. This is consistent with guidelines recommending the dose of vortioxetine be reduced by half if a strong CYP2D6 inhibitor is co-administered (Trintellix Product Monograph. Page 1 of 44, Lundbeck).

**Fig. 2:**
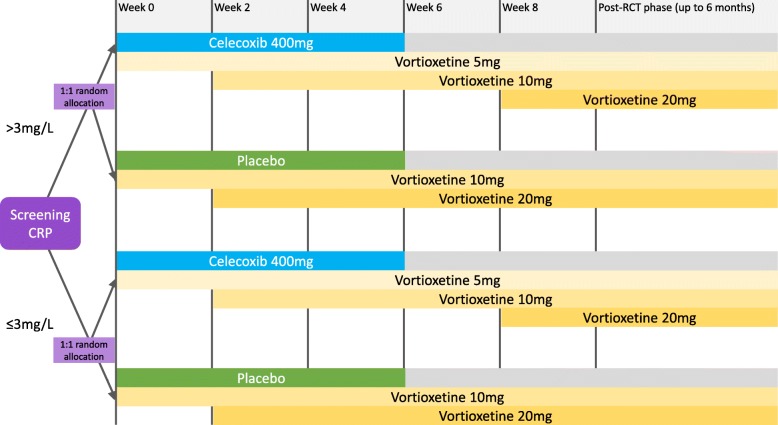
Diagram stating the possible dosages of vortioxetine (antidepressant medication) and celecoxib (anti-inflammatory medication) within each group for all the periods of the trial

Furthermore, dosage of vortioxetine will vary to a small extent between participants even in the same strata, treatment group and time point. This is due to differences in CYP2D6 metabolic functionality in the general population related to common genetic variants [44]. Unfortunately, testing for genetic markers of CYP2D6 dysfunction at baseline, or plasma concentration of vortioxetine in all participants at all necessary time points, is beyond the scope of this study. Nevertheless, we account for this in two ways. First, for the safety and comfort of participants, each person will be started on a low dose and only increase if and when acceptable tolerability is demonstrated, as measured by a low number and mild severity of side effects reported to trial researchers on an in-house side effects questionnaire. The possible doses by strata and treatment group are given in Fig. 2. We are also able to assume, using this method, that the plasma concentration is comparable between all participants throughout the trial [44]. Additionally, the variable of dose differences will be factored into statistical analysis by noting the cumulative total dose of vortioxetine per participant over the entire study.

To promote participant retention, study participants are encouraged to contact the study team if any side effects cause them serious concern between appointments, allowing for the dosage of vortioxetine to be lowered if possible, in the hope of reducing the likelihood of early withdrawal from the study. In the case of early withdrawal from the study, all endpoint scales and blood collection will be conducted immediately for later analysis.

### Double-blinding

The trial psychiatrist and the study PI do not administer the MADRS or any of the functional rating scales, as they are aware of both the dose of vortioxetine and whether a participant is receiving celecoxib or placebo. While the PI is aware which study participants are receiving celecoxib and which participants are receiving placebo, the study rater and participants remain fully blinded during the entire trial. Celecoxib has been encapsulated to have the same appearance as placebo; celecoxib and placebo have been packaged in the same way. A randomisation list has been created by the Royal Adelaide Hospital pharmacy and provided to the trial psychiatrist. The dosage of vortioxetine is not marked specifically on the package provided to participants in order to maintain blinding of participants as well as researchers administering the scales who may see the boxes.

### Study sample size

Given a power of 0.95, and an alpha error of 0.05, the study sample size required to detect a change in MADRS of an effect size of 0.25 between baseline and week 6 would be 50 study participants in each of the four groups. This would be a total of 200 study participants (100 participants in the ‘Depression with inflammation’ stratum and 100 study participants in the ‘Depression without inflammation’ stratum; 50 participants in each treatment group, respectively).

We have found in our internal analyses that around 40–50% of individuals have a CRP level of > 3 mg/L, so we expect to be able to recruit 100 participants to the ‘Depression with inflammation’ stratum and 100 participants to the ‘Depression without inflammation’ stratum. The proposed study duration is two years, which will allow time to recruit the required number of study participants.

### Study schedule

During the screening visit, detailed study information is provided to participants (see Additional file 1) and written informed consent obtained (see Additional file 2). A member of the research team assesses symptoms severity and participant’s eligibility and measures vital signs. Following a blood test, participants are allocated into the ‘Depression with inflammation’ or ‘Depression without inflammation’ strata of the study. Study participants are taken off their regular antidepressant medication after screening and in the week before trial medication is started.

During the baseline visit, demographics (including date of birth, sex and race) and years of education are recorded. In addition, psychiatric and medical history, number of psychotropic medications received, alcohol and nicotine history, vital signs and anthropometric data are collected. A physical examination is also performed during this visit.

According to stratified recruitment into the two treatment strata and the random allocation into two treatment groups, participants in both the ‘Depression with inflammation’ and ‘Depression without inflammation’ stratum receive celecoxib 400 mg daily or placebo. The starting dose of vortioxetine in both study strata is vortioxetine 5 mg daily for those receiving celecoxib, due to possible drug–drug interactions, and vortioxetine 10 mg daily for those receiving placebo. Two weeks after commencement of treatment, participants receiving vortioxetine 5 mg daily and 10 mg daily are provided the opportunity to increase to vortioxetine 10 mg or 20 mg daily, respectively, following an assessment by a research clinician (Fig. 2). The decision to increase the dose or to leave it the same is made by the trial psychiatrist after review of the change in depressive symptoms from baseline according to the MADRS and the presence and severity of side effects, rated by a blinded researcher. The dose of vortioxetine is increased only if the side effects experienced are easily manageable and the participants have not achieved recovery (MADRS score < 7). In study participants reporting symptoms consistent with an excessive plasma concentration of vortioxetine, the trial psychiatrist will reduce the dose of vortioxetine without informing the participant or blinded researchers administering scales. After six weeks of treatment, celecoxib and placebo are ceased, but the antidepressant vortioxetine is continued for at least two weeks. After the completion of the RCT phase, study participants are given the opportunity to continue taking vortioxetine into the post-RCT study phase for up to six months. Following an assessment by a research clinician, study participants who had been taking vortioxetine 5 mg or 10 mg daily during the RCT due to celecoxib co-administration are given the opportunity to increase to vortioxetine 10 mg and 20 mg daily, respectively; otherwise vortioxetine is continued in the post-RCT phase at the same dose as for the RCT phase.

A member of the research team assesses symptoms severity, cognitive function, functional and workplace productivity, and other secondary assessments at baseline visit, fortnightly during the RCT period, and at the three-month and six-month post-RCT visits (see Fig. 1). Participants are asked to report any treatment side effects at each visit. They are also asked to return the vortioxetine packages at each fortnightly visit and receive new ones regardless of whether they have finished a packet or not, so that compliance can be gauged. Similarly, the celecoxib or placebo medication is also returned at the end of six weeks.

**Fig. 3:**
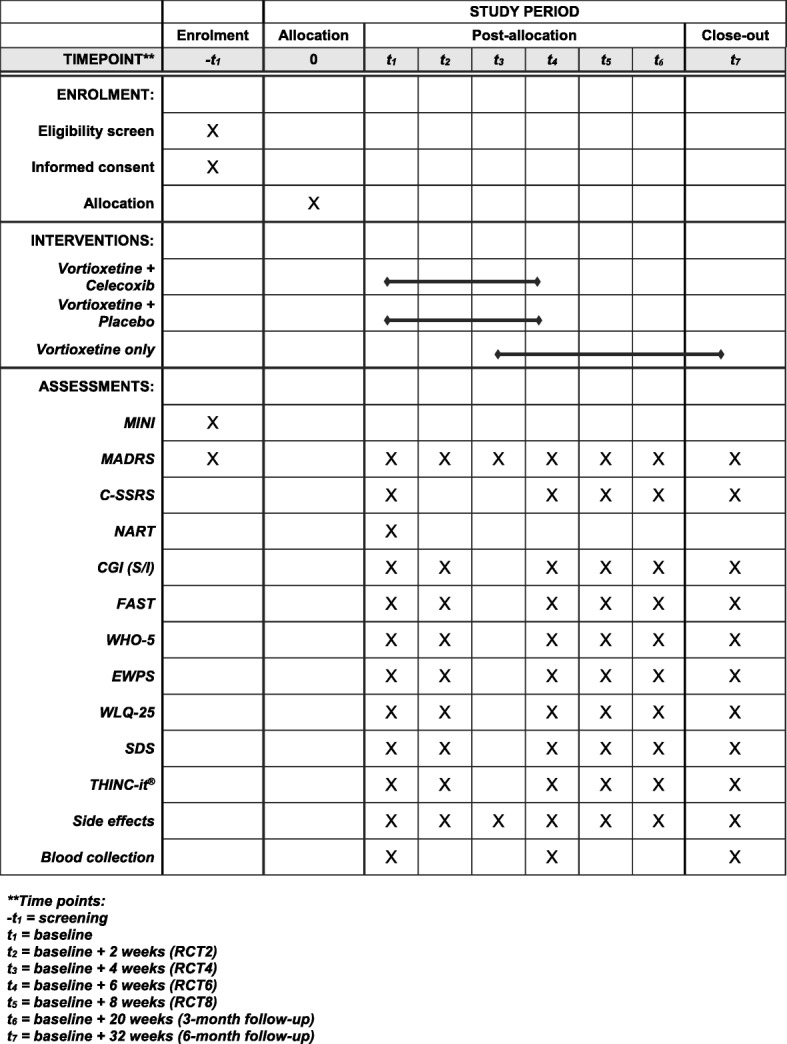
SPIRIT figure. Schedule of enrolment, interventions and assessments

An additional checklist and an additional figure have been provided for further details regarding the study of enrolment, interventions and assessments for the current study (Additional file 3 and Fig. 3).

### Inclusion and exclusion criteria

Participants with MDD are selected according to the following selection criteria: they must be aged 18–75 years; be outpatients in a psychiatric setting with MADRS score ≥ 26 at screening and baseline visits; have at least one prior episode of depression, validated by previous treatment; and the current major depressive episode (MDE) is confirmed by the MINI and has a duration of at least three months. Exclusion criteria are: current alcohol and/or substance use disorder; presence of a co-morbid psychiatric disorder as a focus of clinical concern; primary inflammatory or immune-related disorder; neurodegenerative disorder; history of neurological disorder; past peptic ulcer disease or history of gastrointestinal (GI) bleeding; unstable coronary artery or cardiovascular disease; renal impairment; physical, cognitive, reading, and learning or language impairment. In addition, past hypersensitivity to vortioxetine or to celecoxib and use of concomitant medications able to affect cognitive function or to induce drug–drug interaction are also considered exclusion criteria.

### Data storage and participant confidentiality

Data are stored both on secure, password-protected internal servers and also physically in locked storage space in locked rooms, accessible only to researchers on the trial individually approved by the Ethics Committee mentioned below. Biological specimens are marked with a code unidentifiable to persons unaffiliated with the project. Prepared data reports may be shared with future collaborators but do not contain identifiable participant information. Data will be stored for 15 years after study completion. The results of the trial will be made publicly available via official publication and media release at the trial’s conclusion.

An independent Data Monitoring Committee (DMC) will be formed and comprise experienced local, national and international members.

### Ethics

The study was approved by the Royal Adelaide Hospital Human Research Ethics Committee (HREC; reference number R20170320 HREC/17/RAH/111). Study participants have all study details explained to them in writing and in person by a member of the study team before giving written informed consent. The consent form states that refusal to participate or subsequent withdrawal from the study will in no way influence any treatment that the participant would receive, at the time of the study or in the future. Any and all serious adverse events to occur considered to be related to the trial are reported to the aforementioned HREC by the PI. In the event of participants suffering harm due to the study, the trial is covered by indemnity insurance.

## Medications

### Celecoxib

Celecoxib, a cyclooxygenase 2 inhibitor, is a NSAID that has previously been used as an adjunct to antidepressant medication in the treatment of MDD in four trials, with three of these trials exploring six weeks of treatment at 400 mg daily [45–47] and one trial of eight weeks at 200 mg daily [48]. All four of these trials suggested improved antidepressant treatment effects for celecoxib add-on treatment, compared to antidepressants plus placebo. Recent meta-analyses associated celecoxib with an increased efficacy of antidepressant therapy [41]. In particular, augmentation of antidepressant medication with celecoxib showed an improved antidepressant effect by a standard mean difference (SMD) of 0.82 (95% confidence interval [CI]: 0.46–1.17) and without heterogeneity between the studies (I^2^ = 0%) [41]. Furthermore, remission of depression was improved by an odds ratio (OR) of 7.89 (95% CI: 2.94–21.17) and treatment response by an OR of 6.59 (95% CI: 2.24–19.42) by this 6–8-week add-on treatment hence the choice of celecoxib 400 mg for our study. Finally, one trial included peripheral blood tests and found that higher levels of the pro-inflammatory marker IL-6 predicted better antidepressant response to the celecoxib add-on [45]. In the present study, celecoxib 400 mg or placebo is given daily to all the participants at a constant dosage from baseline to week 6.

### Vortioxetine

Vortioxetine has been chosen as it has a TGA indication in Australia for the treatment of MDD. The Royal Australian and New Zealand College of Psychiatrists (RANZCP) guidelines for the treatment of MDD include use of antidepressant medication such as vortioxetine. The RANZCP guidelines state vortioxetine is most likely to be useful in melancholia and severe depression [39]. Furthermore, vortioxetine is the only antidepressant listed in the RANZCP guidelines that is likely to be useful in enhancing the remittance of cognitive symptoms of depression [39], since it has been demonstrated to have beneficial effects on the cognitive symptoms seen in MDD [40].

All the study medication will be provided for free over the entire RCT and post-RCT periods, for eight months in total.

## Clinical, self-report and cognitive assessment

When not self-administered, the following clinical and cognitive assessment are administered by a clinician researcher who is blind to whether participants receive celecoxib or placebo. The administering researcher has been trained in all clinician-rated scales. These scales have been referred to as ‘clinician-administered questionnaire’ in the text. A summary of all scales used in the study can be found in Table 2 (see Table 2). The table lists whether the rater is a study clinician who is blinded to anti-inflammatory or placebo medication status and baseline CRP level, or self-administered. While many scales are self-administered, they are all completed under the supervision of research personnel who can offer clarification if required.

**Table 2:** Table summarising all clinician-administered and self-administered scales used in the study. Study scales used in the anti-inflammatory treatment of depression study, their range, cutoff for study eligibility and rater identity

Scale name	Purpose of scale	Score range	Study cutoff	Rater
Montgomery–Åsberg Depression Rating Scale (MADRS)	Detect the presence and severity of depressive symptoms	0–60 (0–6 = normal/recovered; 7–19 = mild depression; 20–34 = moderate depression; and 35–60 = severe depression)	≥ 26, or if Item 10’s (suicidal ideation) score ≥ 5	Blinded study clinician
Mini-International Neuropsychiatric Interview (MINI)	Fulfilment or non-fulfilment of minimum diagnostic criteria for Axis I neuropsychiatric disorders and antisocial personality disorder	N/A	Any current psychotic disorder	Blinded study clinician
Columbia-Suicide Severity Rating Scale (C-SSRS)	Relative severity of suicidal ideation and behaviour in the last month and lifetime	0–33, where the higher the number, the greater the severity of ideation and likelihood of suicidal behaviour	N/A	Blinded study clinician
National Adult Reading Test (NART)	Predictor of IQ, independent of psychiatric morbidity	0–50, with higher scores correlated with higher IQs	N/A	Blinded study clinician
Clinical Global Impression scale (CGI)	Allows for quantification of patient morbidity (CGI-S), and progress and treatment response over time (CGI-I)	2 scales of 0–7; in CGI-S, 0 is normal while 7 indicates complete disability; and in CGI-I, 0 indicates substantial improvement while 7 indicates severe exacerbation of symptoms	N/A	Blinded study clinician
Functioning Assessment Short Test (FAST)	The extent of difficulty the participant has had in specific areas of everyday life in the specified timeframe	0–72, where the higher the number, the more severe the difficulty in functioning	N/A	Blinded study clinician
WHO-5 Well-Being Index	Estimates the participant’s well-being by gauging the proportion of time spent in a positive state of mind in the last 2 weeks	0–100, where the higher the number, the greater the participant’s well-being	N/A	Self-administered
Endicott Work Productivity Scale (EWPS)	Assessment of productivity when at work and record of hours worked	0–100, where the higher the number, the poorer the participant’s productivity at work	N/A	Self-administered
Work Limitation Questionnaire (WLQ-25)	Assessment of impairment to full functional capacity at work	0–100, where the higher the number, the less able the participant is to meet the requirements of their work	N/A	Self-administered
Sheehan Disability Scale (SDS)	The degree to which the participant has been impaired in work/school, social and family life due to disability	3 scales from 0 to 10, considered independently or combined; and count of days unable to work or unproductive	N/A	Self-administered
THINC-it® Perceived Deficits Questionnaire-5-D (PDQ-5-D)	Participant’s own recollection of cognitive difficulties in the past 7 days	0–20, where the higher the number, the greater the perceived cognitive deficit; score can also be multiplied by 200 to give the same scoring range as the subsequent THINCit® tests	N/A	Self-administered
THINC-it® Spotter	Test of attention and response speed	Overall score: 0–4000, where a higher number indicates greater ability in all measures; additionally, accuracy (range: 0–40) and speed (mean response time) are recorded	N/A	Self-administered
THINC-it® Symbol Check	Test of working memory and attention	Overall score: 0–4000, where a higher number indicates greater ability in all measures; additionally, accuracy (count correct vs incorrect) and speed (mean response time) are recorded	N/A	Self-administered
THINC-it® Codebreaker	Test of attention, perceptual speed, motor speed, visual scanning and memory	Overall score: 0–4000, where a higher number indicates greater ability in all measures; additionally, accuracy (count correct vs incorrect) and speed (mean response time, maximum 1 s) are recorded	N/A	Self-administered
THINC-it® Trails	Test of visual search speed, scanning, speed of processing, mental flexibility and executive functioning	Overall score: 0–4000, where a higher number indicates greater ability in all measures; additionally, accuracy (count correct vs incorrect, error type) and speed (total completion time) are recorded	N/A	Self-administered

### Diagnostic interview and symptom severity

#### Montgomery–Åsberg Depression Rating Scale (MADRS)

Study participants are evaluated with the MADRS at the screening and baseline visits to assure the score is ≥ 26, according to inclusion criteria and at RCT and post-RCT visits. The MADRS is a frequently used and well-validated clinician-administered questionnaire consisting of 10 items assessing the following depressive symptoms: apparent sadness; reported sadness; inner tension; reduced sleep; reduced appetite; concentration difficulties; lassitude; inability to feel; pessimistic thoughts; and suicidal thoughts. All of the items are rated from 0 to 6. The clinician must decide whether the rating lies on the defined scale steps (0, 2, 4, 6) or between them (1, 3, 5). This clinician-rated scale allows assessment of the severity of depressive symptoms. The total score is in the range of 0–60 and the higher the global score, the more serious the depressive symptoms. It is designed to be sensitive to change resulting from antidepressant therapy [49]. Internal rater consistency is controlled by having all trained clinician researchers complete rating scales based on standard video interviews.

#### Mini-International Neuropsychiatric Interview (MINI)

The diagnosis of MDD is confirmed using the MINI during the screening visit. The MINI is a widely used structured diagnostic interview which enables clinicians to make diagnoses of psychiatric disorder [50, 51]. It is a clinician-administered questionnaire divided into modules corresponding to diagnostic categories (MDD, suicidality, bipolar disorder, panic disorder, agoraphobia, social phobia, obsessive-compulsive disorder, post-traumatic stress disorder, alcohol dependence/abuse, substance dependence/abuse, psychotic disorder and mood disorder with psychotic features, anorexia nervosa, bulimia nervosa, generalised anxiety disorder, antisocial personality disorder). The diagnosis of MDD is confirmed if participants experience five or more of nine symptoms (depressed mood, loss of interest, significant weight loss or gain or decrease or increase in appetite, insomnia or hypersomnia, psychomotor agitation or retardation, fatigue or loss or energy, feelings of worthlessness or excessive or inappropriate guilt, diminished ability to think or concentrate or indecisiveness, recurrent thoughts of death or recurrent suicidal ideation without a specific plan or suicide attempt or a specific plan) including depressed mood or loss of interest.

#### Columbia-Suicide Severity Rating Scale (C-SSRS)

The C-SSRS is a widely used clinician-administered questionnaire for assessment of suicidal ideation or behaviour of participants [52]. It consists of a series of questions assessing suicide ideation, action taken to prepare for suicide and whether participants attempted suicide or began a suicide attempt.

### Functioning

#### National Adult Reading Test (NART)

The NART is used to estimate premorbid intelligence [53]. It consists of a list of 50 irregularly pronounced words in order of increasing difficulty. The participants have to read aloud down this list of words and the number of pronunciation errors is recorded. The clinician has previously been familiarised with all the words before administering the test.

#### Clinical Global Impression scale (CGI)

The CGI scale is an easy tool to quantify patient progress and treatment response over time. It assesses the clinician’s view of patient global functioning at baseline and after initiation of treatment. The CGI has two components: the CGI-Severity, which rates illness severity, and the CGI-Improvement, which rates change from baseline [54].

#### Functioning Assessment Short Test (FAST)

The FAST is a clinician-administered questionnaire referring to the last 15 days before assessment. It consists of 24 items divided among the following six areas of functioning: autonomy; occupational functioning; cognitive functioning; financial issues; interpersonal relationships; and leisure time. All of the items are rated from 0 (no difficulty) to 3 (severe difficulty); the higher the global score, the more serious the difficulties [55].

#### WHO-5 Well-Being Index

The WHO-5 Well-Being Index is a short, self-administered questionnaire that measures current mental well-being of participants. It consists of five items related to mood, vitality and general interests. It is a reliable measure of emotional functioning and a good screening tool for depression [56].

### Work productivity

#### Endicott Work Productivity Scale (EWPS)

The EWPS is a self-administered questionnaire assessing work productivity. It consists of 25 items and measures presenteeism and the frequency of productive behaviours during the previous week. All the items are rated from 0 (‘never’) to 4 (‘almost always’). The score of all scores is computed and the higher the global score, the worse the work productivity [57].

#### Work Limitation Questionnaire (WLQ-25)

The WLQ-25 is a self-administered questionnaire used to measure the impact of MDD on work performance. Is consists of 25 items divided among four areas: time demands; physical demands; mental/interpersonal demands; and output demands. For each item, the participant has to rate the amount of time he/she experienced difficulty (from ‘never’ to ‘always’) in the two most recent weeks [57].

#### Sheehan Disability Scale (SDS)

The SDS assesses functional impairment in the three following domains: work/school; social; and family life. It is a brief self-report tool in which the participant has to rate the extent to which he experienced disability and impairment in those domains from 0 (not at all) to 10 (extremely) during the past week. Additional questions estimate the frequency of unproductivity at school/work [57].

### Cognitive assessment (THINC-it tool)

The THINC-it tool has been developed to screen for cognitive dysfunction in patients with MDD. The tasks are consistently delivered in the following order: Spotter; Symbol Check; Codebreaker; and Trails, with a clinician blinded to treatment group instructing participants to read the tutorial instructions preceding each of the THINC-it tasks [58]. In addition, Perceived Deficits Questionnaire-5-D (PDQ-5-D) is administered to all participants. PDQ-5-D is a five-item auto-questionnaire measuring subjectively reported cognitive deficits.

#### Spotter

The Spotter test is used to assess attention and speed of response. In this test, participants are instructed to respond by pressing keys on the left- or the right-hand side of the keyboard corresponding to the direction to which an arrow on the screen is pointing.

#### Symbol Check

The Symbol Check test measures working memory and attention skills. In this test, the participant is presented a series of stimuli at a constant rate. The task is to map the currently presented stimulus on arrow keys of a keyboard to one they have recently seen in the stream (one position back).

#### Codebreaker

The Codebreaker test, which is modelled on the digit symbol substitution test (DSST), measures attention, perceptual speed, motor speed, visual scanning and memory. It requires the examinee to transcribe a unique geometric symbol with its corresponding number. The examinee is initially shown a key containing the numbers from 1 to 6. Under each number, there is a corresponding geometric symbol. The participant is then shown a series of numbers on the screen and asked to match the number with the corresponding geometric symbol.

#### Trails

The Trails test is designed to test elements of executive function. Participants are instructed to connect a set of 18 dots as fast as possible while still maintaining accuracy. It can provide information about visual search speed, scanning, speed of processing, mental flexibility, as well as executive functioning.

## Blood analysis

A blood test is taken during the screening visit for measuring CRP levels, for the allocation into the ‘Depression with inflammation’ and ‘Depression without inflammation’ strata of the study. Another blood test to measure haemoglobin and a pregnancy test in premenopausal women is performed at the same time. Anti-inflammatory medication is associated with higher risk of GI bleeding [59] and haemoglobin levels have been suggested as a relevant marker of GI damage [60]. Therefore, measuring haemoglobin levels before treatment allows detection of potential pre-existing GI damages. If haemoglobin levels are abnormal, participants are referred to their treating doctor before starting the treatment. The pregnancy test is performed since vortioxetine is classified as a category B3 drug in Australia, meaning that studies in animals have shown evidence of an increased occurrence of fetal damage, the significance of which is considered uncertain in humans. Pregnant women therefore are not eligible for this trial. Blood tests are also performed at the baseline visit, after six weeks of treatment and at the six-month post-trial visit for analyses of serum inflammatory proteins (TNF-α, IL-6 and IL-1β). The blood sample is collected earlier (at last observation) if participants withdraw from the study. The storage of biospecimens is split between different secured freezers at the Adelaide Psychiatric Biomarker Centre at the Discipline of Psychiatry, University of Adelaide, the Adelaide Health and Medical Sciences building, all of which are monitored by a central alert system 24 h/day seven days a week.

## Results analysis

The primary outcome (change in MADRS score from baseline to week 6) will be analysed using a mixed effects model for repeated measurements (MMRM, all available data), with freely varying mean and covariance structures. Treatment will be included as fixed factors. The baseline CRP level and stratified group (‘Depression with inflammation’ versus ‘Depression without inflammation’) will be controlled for. The existence of an interaction between inflammation at baseline and treatment will be assessed, recognising that the study is only powered to detect very large interaction effects. As previously recommended, ANCOVA will be used to account for possible baseline imbalance between treatment arms, thus allowing for participant differences [61]. As previously stated, we hypothesise that a reduction in MADRS score will be higher in participants receiving vortioxetine plus celecoxib in the ‘Depression with inflammation’ stratum compared to participants receiving the same treatment in the ‘Depression with no inflammation’ stratum. The potential treatment effect on CRP will be separated into the stratified strata (‘Depression with inflammation’ versus ‘Depression without inflammation’) as well as for the total group (all four groups). For dichotomous endpoints, such as response and remission, logistic regression with treatment as a factor and the baseline score as a covariate will be used. For continuous endpoints, the same methodology as that described for the primary endpoint will also be used. Continuous endpoints will also be analysed using observed case (OC) and last observation carried forward (LOCF), with LOCF being used to account for missing data and to perform sensitivity analysis. Association between function (FAST, Work Limitations Questionnaire and Sheehan Disability Scale) and endpoints addressing cognitive dysfunction will be explored by means of a partial correlation coefficient. Adverse events and the CSSR-S will be summarised using descriptive statistics.

## Discussion

Abundant literature has highlighted that inflammation is closely associated with depressive symptoms in patients with MDD [6, 13, 24, 62–65]. Targeting inflammation has therefore become a promising strategy in improving depressive symptoms. Interestingly, numerous studies demonstrate a beneficial effect of anti-inflammatory medications (e.g. NSAIDs or anti-cytokines) on depressive symptoms in patients with MDD [34, 36, 38]. Specifically, administering COX-2 inhibitors such as celecoxib in addition to antidepressant treatment for six or eight weeks improves treatment response and remission in comparison to antidepressant medication alone [48].

According to this literature, identifying individuals with depression and inflammation could therefore help in developing new personalised treatment for MDD. However, to our knowledge, there is no previous study assessing the therapeutic effect of administering anti-inflammatory medication combined with antidepressant medication on the basis of raised inflammatory markers at baseline. Through the RCT we propose, we will be able to investigate the effectiveness of using a simple blood test, before treatment of depression, to guide the use of anti-inflammatory medication as an addition to antidepressant medication in patients with depression and inflammation. Indeed, if the efficacy of augmenting vortioxetine with celecoxib 400 mg is higher in participants with inflammation at baseline compared to participants with no inflammation at baseline, it would be recommended to use this augmentation strategy only in MDD patients with raised inflammation before treatment.

The primary outcome of this study will be MADRS score. It is expected that antidepressant combined with anti-inflammatory medication will reduce MADRS score by 50% from baseline to six weeks (end of treatment) in the ‘Depression with inflammation’ stratum of the study. We also expect that the reduction in MADRS score in the ‘Depression with inflammation’ stratum will be larger among those for whom vortioxetine treatment has been augmented with celecoxib than those in the same stratum receiving vortioxetine and placebo. Furthermore, the MADRS score reduction in the former group will also surpass that achieved by both treatment groups in the ‘Depression with no inflammation’ stratum. CRP level as a secondary outcome measure is expected to be reduced by 50% between baseline and six weeks in the group treated with antidepressant and anti-inflammatory medication in the ‘Depression with inflammation’ stratum of the study and is not expected to change in the group treated with antidepressant only in the same stratum. Similarly, CRP levels are not expected to be changed following both treatments in the ‘Depression with no inflammation’ stratum. Secondary outcome measures also include other inflammatory markers (TNF-α, IL-6 and IL-1β), levels which are expected to be reduced by 50%. In addition, a 50% improvement in cognitive dysfunction, functional measures and workplace functioning is expected. Finally, this study also assesses the safety of the anti-inflammatory celecoxib 400 mg when combined with vortioxetine by measuring potential side effects at each visit.

It is hypothesised that primary and secondary outcome measures will not show a statistically significant difference between participants receiving vortioxetine only and participants receiving vortioxetine plus celecoxib within the ‘Depression with no inflammation’ stratum of the study.

In summary, we anticipate that the results of this trial will allow the development of a decision aid for depression medication, thus improving treatment response and remission, especially in patients suffering from recurrent treatment resistant depression.

## Trial status

The trial has commenced recruitment and participant inclusion in November 2017.

If any important changes to the study protocol become necessary, where appropriate, the PI will notify the HREC, trial register, financial sponsor, University and the present journal. The HREC and PI, respectively, have the power to end the study early if the need arises. The trial is subject to audit at any time by the HREC and/or DMC.

## Additional files

Additional file 1: Study information sheet. Detailed study information is provided to participants during the screening visit before written consent form is obtained. (PDF 368 kb)

Additional file 2: Participant consent form. The written informed consent is obtained by the participants before starting the study. It states that refusal to participate or subsequent withdrawal from the study will in no way influence any treatment that the participant would receive, at the time of the study or in the future. (DOCX 65 kb)

Additional file 3: SPIRIT checklist. SPIRIT checklist listing all the items addressed in the current study protocol. (DOCX 49 kb)

## Abbreviations

ANCOVA: Analysis of covariance
CGI: Clinical Global Impression
CNS: Central nervous system
CRP: C-reactive protein
C-SSRS: Columbia Suicide Severity Rating Scale
DSST: Digit symbol substitution test
EWPS: Endicott Work Productivity Scale
FAST: Functioning Assessment Short Test
GI: Gastrointestinal
IFN-α: Interferon-α
IL-1ra: Interleukin-1 receptor antagonist
IL-6: Interleukin-6
LOCF: Last observation carried forward
MADRS: Montgomery–Åsberg Depression Rating Scale
MDD: Major depressive disorder
MDE: Major depressive episode
MINI: Mini-International Neuropsychiatric Interview
MMRM: Mixed model for repeated measurements
NART: National Adult Reading Test
NSAID: Non-steroidal anti-inflammatory drug
OC: Observed case
OR: Odds ratio
PDQ-5D: Perceived Deficits Questionnaire-5D
PI: Principal investigator
RANZCP: Royal Australian and New Zealand College of Psychiatrists
RCT: Randomised controlled trial
SDS: Sheehan Disability Scale
SMD: Standard mean difference
TGA: Therapeutic Goods Administration
TNF-α: Tumour necrosis factor-α
WLQ-25: Work Limitation Questionnaire
