# Supplementary material for: Anti-inflammatory treatment of depression: study protocol for a randomised controlled trial of vortioxetine augmented with celecoxib or placebo
Source: Trials. 2018 Aug 20;19:447. doi: 10.1186/s13063-018-2829-7 (PMC6102899; doi:10.1186/s13063-018-2829-7)
Supplement: Supplementary file 1 — Study information sheet. Detailed study information is provided to participants during the screening visit before written consent form is obtained. (PDF 368 kb) [file 13063_2018_2829_MOESM1_ESM.pdf]

Participant Information Sheet

Title: Anti-inflammatory treatment of inflammation associated depression

Introduction

This research is being conducted by Professor Bernhard Baune, in the Discipline of Psychiatry at The University of Adelaide. The purpose of the study is to investigate the effectiveness of using a simple blood test, prior to treatment of depression, to guide the use of anti-inflammatory medication as an addition to antidepressant medication.

This is a research project and you do not have to be involved. If you do not wish to participate, your medical care will not be affected in any way. In addition, you may withdraw from the project at any time after you have commenced.

You will be given a copy of this Participant Information sheet and the Consent form to keep.

What is the purpose of this research?

There is evidence that raised levels of inflammatory markers in the blood are associated with depression. This study aims to investigate whether the use of a blood test of inflammation levels, prior to antidepressant treatment, can improve treatment of depression by guiding the decision to add anti-inflammatory medication to antidepressant treatment.

The research aims to find out whether adding the anti-inflammatory medication Celecoxib (also known as Celebrex) to the antidepressant medication Vortioxetine (also known as Brintellix) can improve symptoms of depression.

What does participation in this research involve?

You will be participating in a randomised controlled treatment (RCT) study. We put people into groups and give each group either antidepressant medication (Vortioxetine) plus anti-inflammatory medication (Celecoxib), or antidepressant medication (Vortioxetine) plus placebo. You will have a one in two chance of receiving the anti-inflammatory medication.

Screening

To understand whether the study is suitable for you, we need to ask you to complete some questionnaires or tests – this is Visit 1 in the 'Schedule of Visits' table. A blood test is also required, to determine levels of the inflammatory marker C-reactive protein prior to commencing the study.

Treatment period

You will come to the Discipline of Psychiatry, University of Adelaide for all of the study visits. During the 8 weeks RCT period, visits involve questions about your physical and mental health and about your mood and function. During these visits, you will be provided with the study medication and we will monitor you closely for side effects.

The baseline visit and the last treatment visit also involve blood sample collection to measure levels of inflammatory markers.

Post-trial period

There is an option to continue Vortioxetine for 6 months after the RCT study. If you have found Vortioxetine to have been of benefit during the RCT study, and you wish to continue in this 6 months post-trial period, Vortioxetine will be provided to you free of charge. Your mood would also be assessed during this post-trial period, and a blood sample is collected at the 6 month post-trial visit to measure inflammatory markers.

The table below sets out the schedule of visits.

Table 1: Schedule of visits

**Visit number**	**Visit length**
Visit 1 (screening)	2 – 3 hours
Visit 2 (baseline, week 1)	2 – 3 hours
Visit 3 (Day 14, week 2)	2 hours
Visit 4 (Day 28, week 4)	1 hour
Visit 5 (Day 42, week 6)	2 – 3 hours
Visit 6 (Day 56, week 8)	2 – 3 hours
Visit 7: Post RCT baseline visit (Day 63, week 9)	30 minutes
Visit 8: Post RCT 3 month visit (week 22)	2 – 3 hours
Visit 9: Post RCT 6 month visit (week 35)	2 – 3 hours

What is the cost associated with this study?

There are no additional costs associated with participating in this research, nor will you be paid for taking part in the research. However, we can re-imburse you for parking fees or public transport up to $20 per visit. All medication, tests, and medical care required as part of the research project (i.e. during the 8 week trial and the 6 months post RCT trial) will be provided to you free of charge. If you wish to continue taking Vortioxetine after the 6 month post-trial period, this medication can be accessed through your usual treating doctor (cost is approximately $65 per month).

Other relevant information about the research project

The study is planned to run from 2017 until early 2019. Around 200 people will participate in the study. At this stage the study is not being conducted at other sites in Australia, however this may happen at a later date.

What are the alternatives to taking part?

You do not have to take part in this research project to receive treatment at your usual hospital or clinic. You can choose to continue treatment as usual with your treating doctor,

which may involve antidepressant medication and / or psychological therapies. Alternatively, there may be other treatment options available for you, such as other psychological therapies or other antidepressant medications. You can discuss these options with your treating doctor to help you decide whether or not to take part in this research project.

What are the possible benefits of taking part?

We hope to get useful information from this study, to benefit others who experience depression. We cannot guarantee or promise that you will receive any benefits from this study.

What are the possible risks and disadvantages of taking part?

People with depression may experience worsening of the symptoms of depression, including the worsening of (or emergence of) suicidal thoughts. This worsening of depression symptoms (or emergence of suicidal thoughts) can occur in people whether or not antidepressant medication is being taken, and suicidal thoughts can remain until depression symptoms have remitted. If you experience any change in your mood which you feel may be associated with the study, please contact the study doctor as soon as possible.

Side effects of both the antidepressant medication and anti-inflammatory medication are likely to be mild and transient. The following side effects may occur from the medications:

Antidepressant medication (Vortioxetine):

Side effects that can be experienced are similar to those that can be experienced from other common antidepressant medications. These side effects can include gastrointestinal side effects, such as initial nausea, vomiting, and constipation. Other side effects include those such as agitation, dizziness, pruritis (itching) and headaches.

Anti-inflammatory medication (Celecoxib):

Side effects that can be experienced are similar to those that can be experienced from other common anti-inflammatory medications, including anti-inflammatory medications available without a prescription. These side effects include gastrointestinal side effects (such as nausea, abdominal pain, and diarrhoea), dizziness, headaches, and pruritis.

Sedation is an uncommon side effect of both study medications, however if you do experience this as a side effect you should avoid driving and working with heavy machinery, and also notify the study investigators.

In addition, your study doctor will give you the patient information leaflets for Celebrex and Brintellix. Please read these for a full explanation of some of the effects of the drugs and ask your study doctor about any issues which are unclear.

Having blood taken through a needle inserted into a vein can cause some discomfort, bruising, minor infection, or transient bleeding. If this occurs, it can be easily treated.

Interactions of the study medications with alcohol and other drugs

There are some medications that should not be taken together with the study medications. In the screening visit, the investigators will determine whether your current medications are likely to interact with the study medications. Examples of medications that may interact with Vortioxetine are other antidepressants (you should not take another antidepressant, including St John's wort, while you are taking Vortioxetine) and Bupropion. These medications interact by increasing the blood levels of Vortioxetine, so you would be more likely to experience the side effects listed (earlier on this page) for Vortioxetine. Examples of medications that may interact with Celecoxib are other anti-inflammatory medications and Bupropion. Therefore you should not take other anti-inflammatory medications or Bupropion while you are taking Celecoxib, as you would be more likely to experience the side effects listed for Celecoxib.

How to take the study medications

The study medications should be taken as prescribed, and as close as possible to the same time each day. If you forget to take a dose of the medication, you should take the medication the next time it is due. You should notify a member of the research team if you forget to take a dose on more than two consecutive occasions. The researchers will also ask when you attend for visits about whether there have been any days where you have forgotten to take the study medication.

Pregnancy Risks

It is important that study participants in this study are not pregnant or breastfeeding. In females where child-bearing is a possibility, a reliable form of contraception, such as hormonal methods, must be used for the duration of the study. If you are female and become pregnant whilst participating in the research project, you should advise your study doctor immediately. Your study doctor will withdraw you from the research project, as you must not continue in the study if you become pregnant.

Confidentiality and Data Security: what will happen to my study information?

By signing the consent form, you consent to the study doctor and relevant research staff collecting and using personal information about you for the purpose of the research project. Any information obtained in connection with this research project that can identify you will remain confidential. Information will be individually identifiable or coded and will be stored at the University of Adelaide with access only given to research personnel.

Your data will be securely stored within locked cabinets within locked rooms at the University of Adelaide. Data is kept for at least 15 years. Any information obtained during the research project is subject to inspection (for the purpose of verifying the procedures and the data) by the University of Adelaide, or as required by law. In addition to the processes described above, data may otherwise be discoverable through processes of law or for assessing compliance with research procedures. You have a right to access the information collected and stored by

researchers about you. You also have a right to request that any information with which you disagree be corrected.

It is anticipated that results of this research project will be published. In any publication and/or presentation, information will be provided in such a way that you cannot be identified, except with your permission.

Any information obtained for the purpose of this research project that can identify you will be treated as confidential and securely stored. It will be disclosed only with your permission, or as required by law.

Blood samples

A blood sample is taken from you to determine biological information about health and disease characteristics and treatment response. Your blood samples will be stored in a coded form. This means that all information directly indicating your identity will be removed from the labelling and replaced with a code. The University of Adelaide will be able to identify which samples are yours. So if you request for your samples to be destroyed, your study code can be used to identify which blood samples should be destroyed. Any data that is stored on a computer or computer disk will be coded and password protected.

It is possible that the investigators may collaborate with interstate or overseas researchers using your de-identified anonymous blood samples and clinical information. This is because sharing results with other research groups can achieve more meaningful results. If this was to occur, your blood samples will still be coded in a de-identified way, and the University of Adelaide will maintain ownership of all data and samples.

If you withdraw consent from the study, any related biological data already obtained will form part of the research project results unless you notify the research team otherwise in the study consent form. You have a right to ask that any stored specimens should be destroyed, but should be aware that data which has already been derived from those specimens may not be able to be destroyed. Any samples to be destroyed will be disposed of in accordance with state and federal laws, as well as regulations of The University of Adelaide that apply to the disposal of human materials and bio-hazardous waste.

Your donation of blood and interview/questionnaire information together is a valuable resource to researchers because it may be helpful to discover relationships between health history or specific characteristics of conditions, disorders, or behaviours. By giving your consent to participate in the current study, you are giving permission for us to share our findings from this study with other authorized researchers from the Australian and international scientific community.

Could the research be stopped unexpectedly?

The research project may be stopped unexpectedly for a number of reasons. These reasons may include:

- unacceptable side effects of the study medications

- the study medication being shown not to be effective
- decisions made by local regulatory / health authorities

Complaints and compensation

If you suffer any complications, you should contact the study doctor straight away, who will assist you in arranging appropriate medical treatment. If you are eligible for Medicare, you can receive any medical care required to treat the complication, free of charge, as a public patient in any Australian public hospital.

An after-hours number will also be provided to all study participants. You can contact this number 24 hours per day, to speak to a study investigator.

The study has been indemnified by the University of Adelaide. You do not give up any legal rights to compensation by participating in this study.

Who has reviewed this research project?

All research conducted in Australia involving humans is reviewed by an independent group of people, a Human Research Ethics Committee (HREC). The ethical aspects of this research project have been approved by the Royal Adelaide Hospital HREC, and The University of Adelaide HREC.

The research will be conducted according to the NHMRC National Statement on Ethical Conduct in Human Research (2007, updated in May 2015). This statement has been developed to protect the interests of people who participate in human research studies.

Further information and who to contact

The person you may need to contact may depend on the nature of your enquiry. If you require further information concerning the project, or if you have any medical problems which may be related to your involvement in the project (for example, side effects), you can contact the principal study doctor on (08) 8313 7382 or any of the following people.

Clinical contact person:

Principal Investigator	Professor Bernhard Baune
Associate Investigator	Dr Natalie Mills
Associate Investigator	Dr Celia Fourrier
Telephone	(08) 8313 7676
Email	research.psychiatry@adelaide.edu.au

If you wish to speak to someone not directly involved in the study about your rights as a volunteer, or about the conduct of the study, you may also contact the Chairperson, Research Ethics Committee, Royal Adelaide Hospital on 8222 4139. A description of this clinical trial will be available on www.anzctr.org.au, as required by the Ethics Committee. This Web site will not include information that can identify you. At most, the Web site will include a summary of the results. You can search this Web site at any time.
