# Supplementary material for: Anti-inflammatory treatment of depression: study protocol for a randomised controlled trial of vortioxetine augmented with celecoxib or placebo
Source: Trials. 2018 Aug 20;19:447. doi: 10.1186/s13063-018-2829-7 (PMC6102899; doi:10.1186/s13063-018-2829-7)
Supplement: Supplementary file 2 — Participant consent form. The written informed consent is obtained by the participants before starting the study. It states that refusal to participate or subsequent withdrawal from the study will in no way influence any treatment that the participant would receive, at the time of the study or in the future. (DOCX 65 kb) [file 13063_2018_2829_MOESM2_ESM.docx]

**Consent form**  - *Adult providing own consent*

**Protocol Name / Title:** Anti-inflammatory treatment of inflammation associated depression

**Investigators:** Professor Bernhard Baune

Declaration by Participant

1. The nature, purpose and risks of the research project have been explained to me. I understand them and agree to take part.
2. I understand that I may not benefit from taking part in the trial.
3. I understand that, while information gained during the study may be published, I will not be identified and my personal results will remain confidential.
4. I understand that I can withdraw from the study at any stage and that this will not affect my medical care, now or in the future.
5. I understand that I should not be pregnant during the course of this trial.
6. I have had the opportunity to discuss taking part in this investigation with a family member or friend.
7. I have agreed to provide a biological sample and its use has been explained and accepted by me. YES ☐ NO ☐
8. I consent that my personal information (but not my name or address) may be made available to researchers for collaboration in the future. YES ☐ NO ☐
9. I consent to being contacted in the future to participate in depression research projects. YES ☐ NO ☐
10. I understand that my refusal to participate or subsequent withdrawal from the project will in no way influence any treatment that I would receive, now or in the future. YES ☐ NO ☐

Use of data in the event of study withdrawal

If you decide to withdraw from the study, the data collected from you by the research team up until the time you withdraw will form part of the research project results unless you do not want this

☐ I DO NOT agree to the use of data collected from me if I decide to withdraw from the study.

**Name of study participant:**

**Signed:**

**Date:**

I certify that I have explained the study to the study participant / volunteer, and consider that he / she understands what is involved.

**Name of Investigator:**

**Signed:**

**Date:**
